# Production and characterisation of waste tire pyrolytic oil – Investigating physical and rheological behaviour of pyrolytic oil modified asphalt binder

**DOI:** 10.1016/j.heliyon.2023.e12851

**Published:** 2023-01-09

**Authors:** Humeyra Bolakar Tosun

**Affiliations:** Civil Engineering, Faculty of Engineering, Aksaray University, Aksaray, Turkey

**Keywords:** Asphalt binder, BBR test, Pyrolytic oil, GC-MS, Rheological properties

## Abstract

The effort of basic restrict the use of petroleum-based products, the use of recycling and alternative resources have tremendous gained interest in the last decades. In this experimental study, waste tire granules were pyrolyzed under a vacuum atmosphere. Pyrolysis experiments occurred in a ﬁxed bed reactor at 450 °C. The chemical characterisation of the pyrolytic oil was exanimated by various analytical methods, such as Infrared spectroscopy (FT-IR), ultimate and gas chromatography coupled to mass spectrometry (GC-MS). It was determined that mainly contains aliphatic and aromatic compounds. In addition, pyrolytic oil was used as a partial replacement for conventional asphalt (petroleum-based) to limit the use of conventional asphalt in the applications of road engineering. The effect of the pyrolytic oil additive on the fundamental physical behaviours of the asphalt was evaluated, and also rheological behaviour of the asphalt binder was investigated by the viscosity (RV) and bending-beam-rheometer (BBR) tests. The addition of pyrolytic oil affected some physical properties of asphalt binders more or less. It was found that conventional and pyrolytic oil-modified asphalt samples met the technical requirement based on BS EN 14771 standard. Finally, it can be concluded that asphalt modified with pyrolytic oil can be used in road engineering applications under low-temperature conditions.

## 1 Introduction

The fast depletion of petroleum resources and increasing environmental concerns have led to intensive research for renewable energy and suitable recycling technologies. European Tyre and Rubber Manufacturers Association (ETRMA) stated that at least 300 million tires in the European zone were produced annually, this was equivalent to about 20% of the world market. In other words, it is commonly accepted that for each tire sold another takes part of the waste, over 1 billion tires achieve the end of their life cycle all over the world annually. These wastes are made use of different fields such as civil engineering applications, stock, energy recovery and recycling [[1], [2], [3], [4]].

The waste is non-degradable as biologically. It needs over 1000 years to decompose in nature completely, therefore this waste leads to a severe threat to the ecology throughout the world [5]. The waste can be both re-used and re-cycled. In the last years, studies are carried on to explore new methods to re-cycle waste tires including re-treading, crumbling, grinding, combustion and pyrolysis [6].

The pyrolysis process, which decomposes materials at high temperatures under an inert atmosphere, turns waste tires into gas, liquid and char products. These products can be used by various industries like the chemical, energy and transportation industries [[7], [8], [9], [10]].

There are many reports in the literature related to the physical, thermal, and chemical properties of pyrolytic oil and its application of use in various industrial processes [[11], [12], [13], [14], [15], [16], [17]]. The most reported is that chemically, pyrolytic oil is constituted by paraffin, olefins, and aromatics. Moreover, the relevant physical properties (density, viscosity, pH value, etc.) and the heating value of pyrolytic oil are like those of diesel [[18], [19], [20], [21], [22], [23]].

Crude oil is converted into useful raw materials for the energy and chemical industries. The refining of petroleum is produced from crude oil including syngas, gasoline, diesel, the fuel of jet, heavy fuel-oil, lubricating oil, paraffin wax, bitumen (asphalt) and coke. Asphalt is used as a crucial raw material in civil engineering applications in a matter of road engineering [[24], [25], [26]]. Although many types of materials such as carbon nano-fibre [27], polymers [[28], [29], [30], [31], [32], [33], [34]], nanoparticles [[35], [36], [37], [38]] and others [[39], [40], [41]], were extensively examined to enhance the features of asphalt in previous studies, there have been relatively few studies were examined the effect of substitution level of pyrolytic oil on the rheological characteristics of asphalt.

In this experimental study, vacuum pyrolysis of the waste tire was performed at 450 °C. The pyrolytic oil was analyzed by some chemical characterization methods such as FT-IR, ultimate and GC-MS. Then, the oil was used as a partial replacement for asphalt to limit the use of petroleum-based (conventional) asphalt in road engineering applications. The effect of the pyrolytic oil replacement on the fundamental physical and rheological behaviour of the binder was examined.

## 2 Material and methods

### 2.1 Material

Granules of waste tire particle sizes of about 20 mm were used as a source of raw materials. It was collected from local tire dealers in Aksaray city, Turkey. The proximate analysis was conducted with the standard method ASTM D3172-73. The fixed-carbon (FC) yield of waste tire was calculated by the difference. The ultimate test was conducted by a Leco CHNS-932 analyzer.

In this study, bitumen with a penetration class of 50/70 supplied from the TÜPRAŞ Refinery was used as an asphalt material. Table 1 is presented some physical characteristics of conventional asphalt (CA).

**Table 1:** The physical characteristics of the CA.

Penetration (at 25 °C, 100 g, 5 s, 0.1 mm)	62
Softening point (°C)	49.2
Flashpoint (°C)	260
Viscosity (at 135 °C) (Pas)	0.280

### 2.2 Methods

#### 2.2.1 Pyrolysis experimental apparatus and procedure

**Fig. 1:**
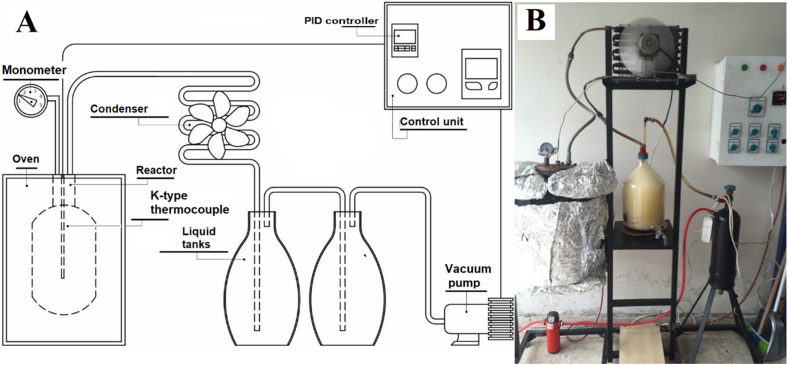
A sketch of the pyrolysis equipment (A) and its picture (B).

The pyrolysis run was performed at 450 °C in a vacuum atmosphere in a fixed-bed reactor equipped with an oven (heating through electrical resistance), liquid collectors, a condenser, a vacuum pump and other components. A sketch of the pyrolysis equipment and its picture is displayed in Fig. 1. The reactor was formed of stainless steel (240 mm-id × 360m-high). Throughout the pyrolysis run, the pyrolysis temperature was operated using a proportional integral derivative (PID). The pyrolysis temperature was measured every minute inside the bed using a K-type thermocouple. The vacuum pressure was adjusted at nearly 10 kPa. According to relative literature, the maximum yield of pyrolytic oil is about 40–55 wt% at a temperature in the range of 400–550 °C. Thus, the selected temperature of 450 °C was suitable for a pyrolysis process [[42], [43], [44], [45]]. In a typical pyrolysis run; a 2000 g sample of the waste tire was taken into the reactor. The target temperature was 450 °C with a heating a rate of approximately 10 °C/min., kept for 40 min The oil was collected by condensation in two condensers combined with the reactor exit.

#### 2.2.2 Analytical methods

The pyrolytic oils were characterized by some characterization methods such as FT-IR, ultimate and GC-MS analysis. An FT-IR spectrometer (model- Alpha FT-IR) was employed to determine the functional groups of the pyrolytic oil. Each FT-IR spectrum was scanned in the region 4000–400 cm^−1^. The ultimate analysis was performed by a Leco CHNS-932 analyser to identify CHN elemental distribution of the oil sample. The pyrolytic oil was characterized by GC-MS (Agilent 6890), to identify chemical compounds in the oil. The pyrolytic oil was mixed for homogenization and filtered with a 0.60 μm filter, prior to GC-MS analysis. The gas chromatograph was equipped with an HP-5 (30 m × 0.25 mm i.d. × film thickness 0.25 μm) capillary column. Helium was used as a non-condensable carrier gas at a flow rate was 1.2 ml/min. The oven was set at a temperature starting at 40 °C, kept for 10 min, then, raised at a rate of 2 °C–170 °C, was held for 5 min, after that the temperature was increased to 250 °C at a holding time of 15 min, then the temperature increased in 320 °C at a heating rate of 15 °C, held for the same temperature for 10 min, The injector temperature was 250 °C and injections were performed in split mode. (a split ratio of 1:10). The end of the column was connected directly to the ion source of an Agilent 5973 series mass selective detector utilised with electron impact ionization mode. The peaks were identified from the NIST MS library.

#### 2.2.3 Asphalt sample preparation

**Fig. 2:**
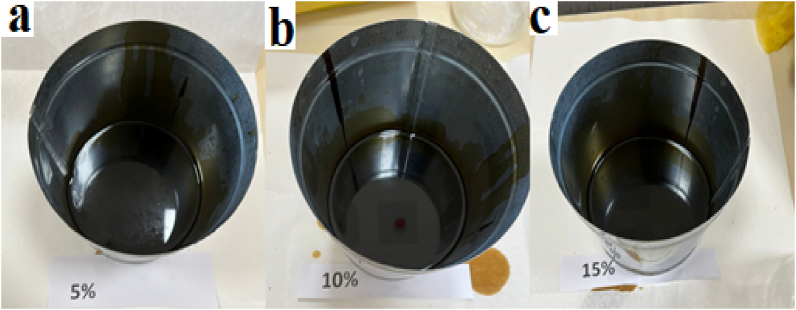
Photographs of asphalt modified with pyrolytic oil (a: 5%, b:10%, c:15%).

The selected pyrolytic oil contents of 5%, 10% and 15% by total weight of the mix were used in this study, this selection was based on the preliminary study and previous literature [46,47]. The procedure for the modification of asphalt is as follows. First, place 50/70 grade bitumen into a 150 °C oven and confirm the bitumen can flow; then, pour the fluid bitumen into a metal pot, mixing the pyrolytic oil in the asphalt for 60 min at 150 °C. The blending rate carried out in this research was 500 r/min. The mixtures were removed from the oven and it was cooled at ambient temperatures of 20 and 25 °C, prior to experiments and analysis. Fig. 2 shows photographs of asphalt modified with pyrolytic oil.

#### 2.2.4 Asphalt characterizations

Physical properties of asphalt samples including penetration test, ductility test, and softening point test were performed to detect the physical performance of the asphalt binders. These tests are commonly known as the conventional physical tests of asphalt binders [[48], [49], [50]]. The penetration test determines the consistency of the bitumen. The test followed ASTM D 5 EN 1426. Penetration is expressed as the amount into which a bitumen sample will penetrate vertically under the specified conditions of temperature, load and loading time of a standard needle. The temperature at which the bitumen reaches the degree and becomes a flowing liquid is expressed as a softening point. The test followed ASTM D 36. In general, a high softening point is an indicator of sensitivity to low temperatures and this feature is preferred in hot climates. The ductility properties of conventional and pyrolytic oil-modified asphalts were measured by a Strassentest instrument at 25 °C based on the criteria of ASTM D 113-86. The rotational viscosity (RV) of the asphalt samples was determined by Bohlin Instruments (Bohlin CS-50) at 135 °C based on ASTM D 4402.

All samples were exposed to short-term ageing using a rolling thin film oven (RTFO) according to EN 12607-1. Long-term ageing to which the sample was subjected through its service life was examined by a pressure ageing vessel method (PAV) based on procedure EN 14769.

The Bending Beam Rheometer (BBR) test was also performed using BBR equipment in following EN 14771 standard.

## 3 Results and discussions

### 3.1 Characterization of waste tire

Table 2 shows the proximate and elemental analysis results of the waste tire; findings are indicated in percentage form by sample weight.

**Table 2:** Proximate and ultimate analysis of waste tire.

Proximate Analysis (Weight %)				Ultimate Analysis (Weight %)				
Moisture	Ash	Volatile Matter	Fixed Carbon	C	H	O	N	S
0.2	12.8	62.2	24.8	84.1	7.3	6.2	0.6	1.8

Proximate and ultimate analyses of waste tyres stated by some authors are shown in Table 3. The difference in findings could be based on the manufacturers’ formulation of the various components of tyres [51].

**Table 3:** Proximate and ultimate analysis of waste tire obtained from the literature.

Characteristics	Reference 1 [52]	Reference 2 [18]	Reference 3 [53]	Reference 4 [54]
Moisture content (%)	1.2	0.7	0.4	
**Proximate analysis (%)**				
Volatiles	61.3	65.0	69.9	73.9
Ash	5.2	4.2	2.1	4.3
Fixed carbon	33.5	30.1	27.5	21.8
**Ultimate analysis (%)**				
C	85.2	83.9	82.3	89.2
H	7.3	6.8	7.3	7.7
O	0.5	–	–	
N	0.4	0.8	0.3	0.5
S	2.3	0.9	1.1	

### 3.2 Analysis of the pyrolytic oil

**Fig. 3:**
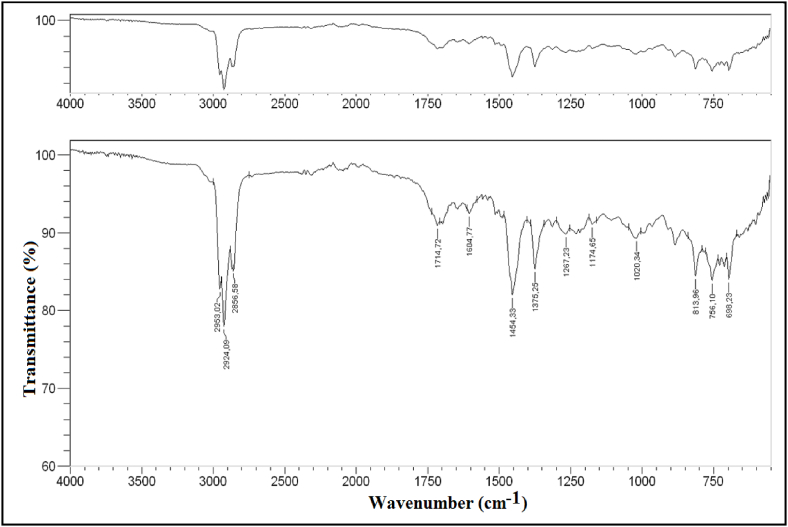
FT-IR spectra of pyrolytic oil.

The FT-IR transmittance spectrum is displayed in Fig. 3. The presence of aromatics compounds is observed at about 3050 cm^−1^ with C–H stretching vibrations. The peaks between 2850 and 300 cm^−1^ C–H stretching vibrations; peaks between 1300 and 1450 cm^−1^ could be ascribed to the existence of alkanes. The peaks ranging from 1780 to 1650 cm^−1^ represent the C=O stretching vibration can be indicated by the presence of aldehydes and/or ketones. The peaks between 1600 cm^−1^ and 815 cm^−1^ attributed to C=C stretching vibrations that could be indicative of alkenes and aromatics. Previous studies have indicated that pyrolytic oil mainly contains aromatic and aliphatic compounds [19,55,56].

The ultimate test of the pyrolytic oil is given in Table 4. Pyrolytic oil had low oxygen contents than raw material. The reduction in oxygen contents of the oil (3.8 wt%) compared to waste tire (6.2 wt%) is significant since it can be used as fuel.

**Table 4:** Ultimate test of waste tire pyrolysis oil.

Ultimate test (Weight %)				
C	H	O	N	S
86.2	8.9	3.8	0.2	0.9

Table 5 depicts the GC-MS analysis findings of pyrolytic oil. The aromatics hydrocarbon compounds found in the oil were all single-ring compounds including benzene and its derivatives, olefins terpenes and PAH. Laresgoiti and co-workers asserted that major compounds in pyrolytic oil included methyl- and ethyl benzene, limonene, and dimethylcyclohexene [9]. Also, Abedeen and co-workers stated that pyrolytic oil is a complex mixture that contains both aliphatic and aromatic hydrocarbons [56]. The identified compounds were consistent with past studies on the chemical composition of pyrolytic oil [[57], [58], [59], [60]].

**Table 5:** Chemical components of pyrolytic oil.

No	RT (min)	Compounds	Area (%)	Category
1	3.64	2-Methyl-benzene	10.78	Benzene
2	3.76	Methylene-cyclohexane	1.24	Alkanes
3	3.82	2-Pentenoic acid	1.11	Acids
4	3.94	3-Methyl-2,4-hexadiene	1.68	Olefins
5	5.84	4-Ethyl-cyclohexene	2.58	Olefins
6	7.31	Ethyl-benzene	2.84	Benzene
7	8.44	1-Ethynyl-1-cyclohexene	6.24	Olefins
8	12.98	1,2 Dihydrocatechol	1.54	Aldehydes
9	13.14	1,2,3-Trimethyl-benzene	11.68	Benzene
10	14.64	1-Ehyl-2-methyl-benzene	3.44	Benzene
11	15.15	d-Limonene	16.46	Terpene
12	15.34	1-Isopropenyl-4-methyl-1,3-cyclohexadiene	4.23	Olefins
13	16.05	1-Methyl-1,2-propadienyl- benzene	2.60	Benzene
14	20.14	2,4-Dimethylstyrene	1.86	Benzene
15	21.84	1-Methyl-1,2-propadienyl- benzene	3.24	Benzene
16	22.64	1,3-Cyclopentadiene, 5-(1-methylethylidene	10.88	Benzene
17	23.85	1,4-Methanonaphthalene	1.84	PAH
18	23.88	1,3-Cyclopentadiene, 5-(1-methylethylidene)	11.65	Benzene
a: obtained at 450 °C				

### 3.3 Physical behaviours of modified asphalt

The physical behaviours of conventional asphalt and modified asphalt with pyrolytic oil are given in Table 6. The physical behaviours of asphalt were affected by the addition of pyrolytic oil: the penetration of asphalt-modified with the pyrolytic oil was determined to be higher than that of the conventional asphalt binder (61), The penetration of the asphalt binder modified with 5, 10 or 15 wt% pyrolytic oil was found to be 217, 241, and 280, respectively. The softening point of asphalt modified pyrolytic oil decreased with a slight increase in pyrolytic oil content. This outcome was shown that the incorporation of pyrolytic oil could lead to a decrease in the high-temperature condition performance of asphalt. When compared to the conventional asphalt binder, the ductility of modified asphalts with 5%, 10%, and 15% pyrolytic oil content increased by 74, 91, and 126, respectively. Chen and co-workers reported that a high ductility value could be improved the low temperature achievement of asphalt, and enhance the low temperature, anti-cracking capability of asphalt before RTFO-aging [61].

**Table 6:** The physical behaviours of conventional asphalt and modified asphalt.

Asphalt binder	Conventional asphalt (CA)	%5 Pyroltic oil + CA	%10Pyroltic oil-CA	%15 Pyroltic oil + CA
Penetration (25 °C)	61	217	241	280
Softening point (°C)	46	37	30	24
Ductility (5 °C, cm)	59	74	91	126

**Fig. 4:**
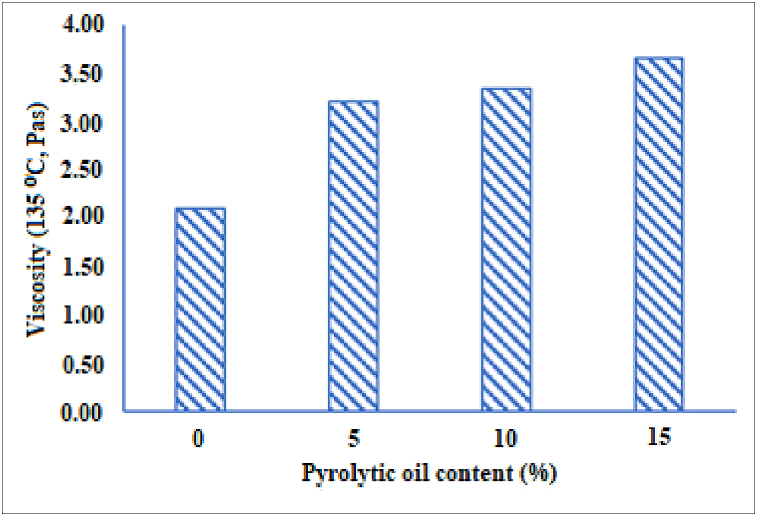
The viscosity of asphalt with different pyrolytic oil contents.

The rotational viscosity (RV) test (at 135 °C) was conducted in this experimental work to determine the mixable and workable behaviours of asphalt binder. Fig. 4 shows the RV values of asphalt with different pyrolytic oil contents (5%,10%, and 15%). The RV values of the asphalts modified with pyrolytic oil were found to be higher than conventional asphalt and gradually increased with the pyrolytic oil replacement rising. The low molecular weight fraction of pyrolytic oil may be responsible for the increased viscosity of the modified asphalt samples. In a similar study, Al-Sabaeei and his co-workers reported that higher pyrolytic oil content leads to lower asphalt viscosity [62].

**Fig. 5:**
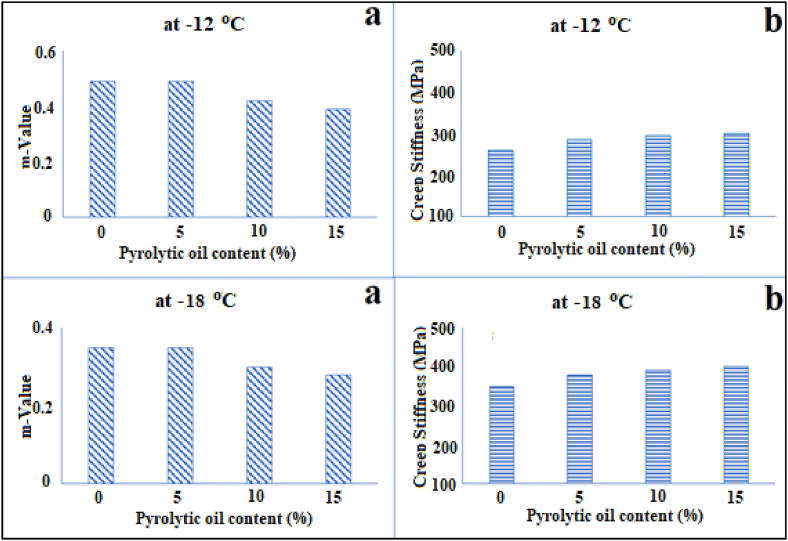
Effect of pyrolytic oil content on creep stiffness (a) and m-value (b) of the BBR tests.

The low temperature conditions performances of modified-asphalt and conventional asphalt were examined using the BBR test method. Fig. 5 presents the BBR results of the PAV-aged asphalt modified with different pyrolytic oil contents. The BBR test results demonstrated that the m-value of modified-asphalt decreased with the reduction of temperature (Fig. 5a). It can be reported that the low temperature ductility and fatigue stability of modified asphalt may be decreased by the rising incorporation of pyrolytic oil. Furthermore, it was found that conventional and modified asphalt samples met the technical requirements, which are creeping stiffness (max. 300 MPa) and m-value (min. 0.3) of BS EN 14771 standard for the specification of the flexural creep stiffness (Fig. 5b). Therefore, it can be concluded that asphalt modified with pyrolytic oil can be used in road engineering applications under low-temperature conditions. Kebritchi and co-workers studied the BBR properties of heavy fraction pyrolytic oil-modified asphalt binders containing warm mixed asphalt additives. They obtained the oil was obtained from vacuum pyrolysis of the waste tire at 600 C. They reported that modified asphalt samples had very good low-temperature flexibility by the BBR test proposing flexible behaviour in winter [63].

## 4 Conclusions

The main conclusions obtained from this experimental study are the following:

• The pyrolytic oil mostly contains aliphatic and aromatic compounds that were identified by FT-IR analysis.
• The ultimate analysis result indicated that the pyrolytic oil has low oxygen content than raw material (waste tire). This result is important since it can be used as a liquid fuel and/or chemical feedstock.
• The GC-MS analysis determined that contains aliphatic and aromatic hydrocarbons
• The addition of pyrolytic oil was affected by the physical properties of asphalt binders. With an increase in pyrolytic oil content, penetration and ductility values of asphalt binders increased, while the softening point of pyrolytic oil modified asphalt decreased with an increase in the oil content.
• The RV of the asphalt modified with pyrolytic oil was found to be higher than conventional asphalt and gradually increased with the pyrolytic oil replacement rising. It can be assumed that a lighter hydrocarbon fraction (lower molecular weight hydrocarbon) in pyrolytic oil was responsible for the high viscosity of the modified asphalt samples. The high molecular weight fraction of pyrolytic oil can be used for asphalt modification for better performance.
• The BBR value for modified asphalt had higher creep stiffness and lower m-values compared to conventional asphalt. They were satisfied with the minimum requirements of BS EN 14771 standard specification for the determination of the flexural creep stiffness of the asphalt binder.

The next studies are recommended to investigate the rheological and physical behaviours of asphalt binders modified with pyrolytic oil obtained from the pyrolysis of waste plastics. It is also recommended that a combination of nano-carbon tubes can be evaluated for modified asphalt binders.

## Data availability

The data that has been used is confidential.

## Conflicts of interest

There are no conflicts of interest.

## Funding statement

This research did not receive any specific grant from funding agencies in the public, commercial, or not-for-profit sectors.
